# Host stress and immune responses during aerosol challenge of Brown Norway rats with *Yersinia pestis*

**DOI:** 10.3389/fcimb.2012.00147

**Published:** 2012-11-30

**Authors:** Susan T. Gater, Kristen N. Peters, Andrew G. Kocsis, Miqdad O. Dhariwala, Deborah M. Anderson, Paul E. Anderson

**Affiliations:** ^1^Laboratory for Infectious Disease Research, University of MissouriColumbia, MO, USA; ^2^Department of Veterinary Pathobiology, University of MissouriColumbia, MO, USA; ^3^Department of Molecular Microbiology and Immunology, University of MissouriColumbia, MO, USA

**Keywords:** *Yersinia pestis*, pneumonic plague, aerosol, Brown Norway rat, infectious disease, stress, corticosterone

## Abstract

Inhalation exposure models are becoming the preferred method for the comparative study of respiratory infectious diseases due to their resemblance to the natural route of infection. To enable precise delivery of pathogen to the lower respiratory tract in a manner that imposes minimal biosafety risk, nose-only exposure systems have been developed. Early inhalation exposure technology for infectious disease research grew out of technology used in asthma research where predominantly the Collison nebulizer is used to generate an aerosol by beating a liquid sample against glass. Although infectious aerosol droplets of 1–5 μm in size can be generated, the Collison often causes loss of viability. In this work, we evaluate a gentler method for aerosolization of living cells and describe the use of the Sparging Liquid Aerosol Generator (SLAG) in a rat pneumonic plague model. The SLAG creates aerosols by continuous dripping of liquid sample on a porous metal disc. We show the generation of 0.5–1 μm *Yersinia pestis* aerosol particles using the SLAG with spray factors typically ranging from 10^−7^ to 10^−8^ with no detectable loss of bacterial viability. Delivery of these infectious particles via nose-only exposure led to the rapid development of lethal pneumonic plague. Further, we evaluated the effect of restraint-stress imposed by the nose-only exposure chamber on early inflammatory responses and bacterial deposition. Elevated serum corticosterone which peaked at 2 h post-procedure indicated the animals experienced stress as a result of restraint in the nose-only chamber. However, we observed no correlation between elevated corticosterone and the amount of bacterial deposition or inflammation in the lungs. Together these data demonstrate the utility of the SLAG and the nose-only chamber for aerosol challenge of rodents by *Y. pestis*.

## Introduction

Inhalation exposure is the best way to model infection of the lower respiratory tract which can have a high fatality rate due to limited immune defense mechanisms (Eisele and Anderson, 2011). In humans, infectious aerosols are created by coughing and sneezing, allowing for the generation of force sufficient to dislodge particles from infected lungs. In the laboratory, modeling of respiratory infections using inhalation exposure technology has favored the use of the Collison nebulizer as a method for aerosolization in the laboratory, originally developed for asthma research (May, 1973). The Collison generates aerosols by forcibly beating liquid culture against glass generating infectious aerosol particles of around 1 μm in diameter (Thomas et al., 2009). Exposure chambers involving unrestrained whole-animal or heavily restrained nose-only challenges are becoming widely used in combination with the Collison nebulizer to deliver a defined and highly reproducible amount of infectious agent. Unfortunately, some bacterial agents such as *Yersinia pestis* and *Pseudomonas aeruginosa*, are sensitive to aerosolization through the Collison nebulizer resulting in the death of 50% or more of the bacterial population (Welkos et al., 2002; Agar et al., 2009). Moreover, even for those pathogens that are less sensitive to the shear forces generated by the Collison, there are likely changes in bacterial gene expression that may otherwise not occur during a natural infection. In fact, virulence gene expression changes due to the stress of liquid shear have been documented, which in turn may impact disease or immunity (Castro et al., 2011). Further, respiratory pathogens such as *Y. pestis* infect humans and impose a significant biosafety risk to the experimental model which can be of particular concern with whole animal exposure chambers.

In order to prevent unknown effects on the infection and to better mimic the natural infection, we explored the use of an alternative nebulizer based on Sparging Liquid Aerosol Generation (SLAG) wherein aerosols are generated by bubbling compressed air through a bacterial suspension onto a metal frit (Mainelis et al., 2005; Simon et al., 2011). In this work, we describe the use of the SLAG for the nose-only inhalation exposure of Brown Norway rats to *Y. pestis*, the causative agent of pneumonic plague.

Pneumonic plague is a rapidly progressing and contagious respiratory infection that leads to death in humans and most mammals within days of infection (Pollitzer, 1954). The bacteria overwhelm the innate immune system of the host due to their rapid growth, evasion of phagocytosis, and induction of necrotic death of inflammatory cells (Bergsbaken and Cookson, 2009). The infection is established following an initial active suppression of the immune response that presumably allows the organism to gain the upper hand (Lathem et al., 2005). A number of virulence factors, including the type III secretion system and secreted proteases are involved in establishing this condition. Vaccines targeting the type III secretion system have been found efficacious in animal models of plague and are under evaluation for use in humans (Rosenzweig et al., 2011b). Because of their similar response to *Y. pestis* infection, rats have been used as a surrogate to evaluate vaccines and therapeutics against plague (Anderson et al., 2009; Rosenzweig et al., 2011a). Whole-animal and nose-only aerosol exposure have been developed for mice, rats, and non-human primates for evaluation of vaccines and therapeutics against pneumonic plague (Davis et al., 1996; Agar et al., 2009; Rosenzweig et al., 2011a; Fellows et al., 2012). Though these systems offer many similarities to the natural infection, animal restraint is generally necessary, especially in nose-only systems where precise delivery to the respiratory tract can occur while minimizing contamination of the animal fur.

Mammals, including rats, respond to stress by the rapid production of corticosteroids. Animal restraint causes an elevation in serum corticosterone that peaks 2 h post-restraint and can be affected by many factors including time of day and method of blood collection (Fagin et al., 1983; Paskittia et al., 2000; Moldow et al., 2005; Vahl et al., 2005; Abatan et al., 2008). Moreover, both anti- and pro-inflammatory responses to stress have been reported (Robert and Kupper, 1999; Soderholm et al., 2002; Vicario et al., 2010). Whether this also occurs as a localized inflammatory response during an inhalation exposure procedure is unknown. An increase in stress in animals under restraint during nose-only inhalation exposure might therefore lead to non-specific inflammatory responses that could influence the developing infection thereby making data generated in these animal models subject to misinterpretation.

In this work, we use the SLAG nebulizer to model pneumonic plague and address the role of restraint-stress in the Brown Norway rat during the early stage of infection. We found that most, but not all, of the animals experienced elevated corticosterone as a result of their restraint in the nose-only chamber. Repeated restraint in the chamber did not prevent an increase in corticosterone and there remained a similar percentage of animals that showed no evidence of stress. We monitored bacterial deposition and Ly6G^+^ cell populations during the early stage of infection and found no correlation between these inflammatory cells and elevated corticosterone levels in the serum and lethal pneumonic plague developed within 72 h. Together, our data demonstrate the utility of the SLAG nebulizer in pneumonic plague models and show that in spite of a temporary stress response, the nose-only challenge did not detectably alter the progression of plague.

## Materials and methods

### Bacterial strains

*Y. pestis* CO92, a clinical isolate of the *Orientalis* biovar, was routinely grown fresh from frozen stocks and streaked for isolation onto heart infusion agar (HIA) plates containing 0.005% Congo Red and 0.2% galactose to identify bacteria that retained the pigmentation locus (Surgalla and Beesley, 1969; Welkos et al., 1997). For pneumonic plague challenge, 6 flasks of 50 ml HIB supplemented with 2.5 mM CaCl_2_ were each inoculated with a single red pigmented colony and grown for 24 h at 37°C, 120 rpm. Bacteria were collected by centrifugation, washed once, and re-suspended in sterile PBS and pooled and the OD_600_ was measured. As necessary, bacteria were diluted in sterile PBS to achieve a desired OD_600_. All handling of samples containing live *Y. pestis* CO92 was performed in a select agent authorized biosafety level 3 laboratory under protocols approved by the University of Missouri Institutional Biosafety Committee.

### Aerosol generation

**Figure 1:**
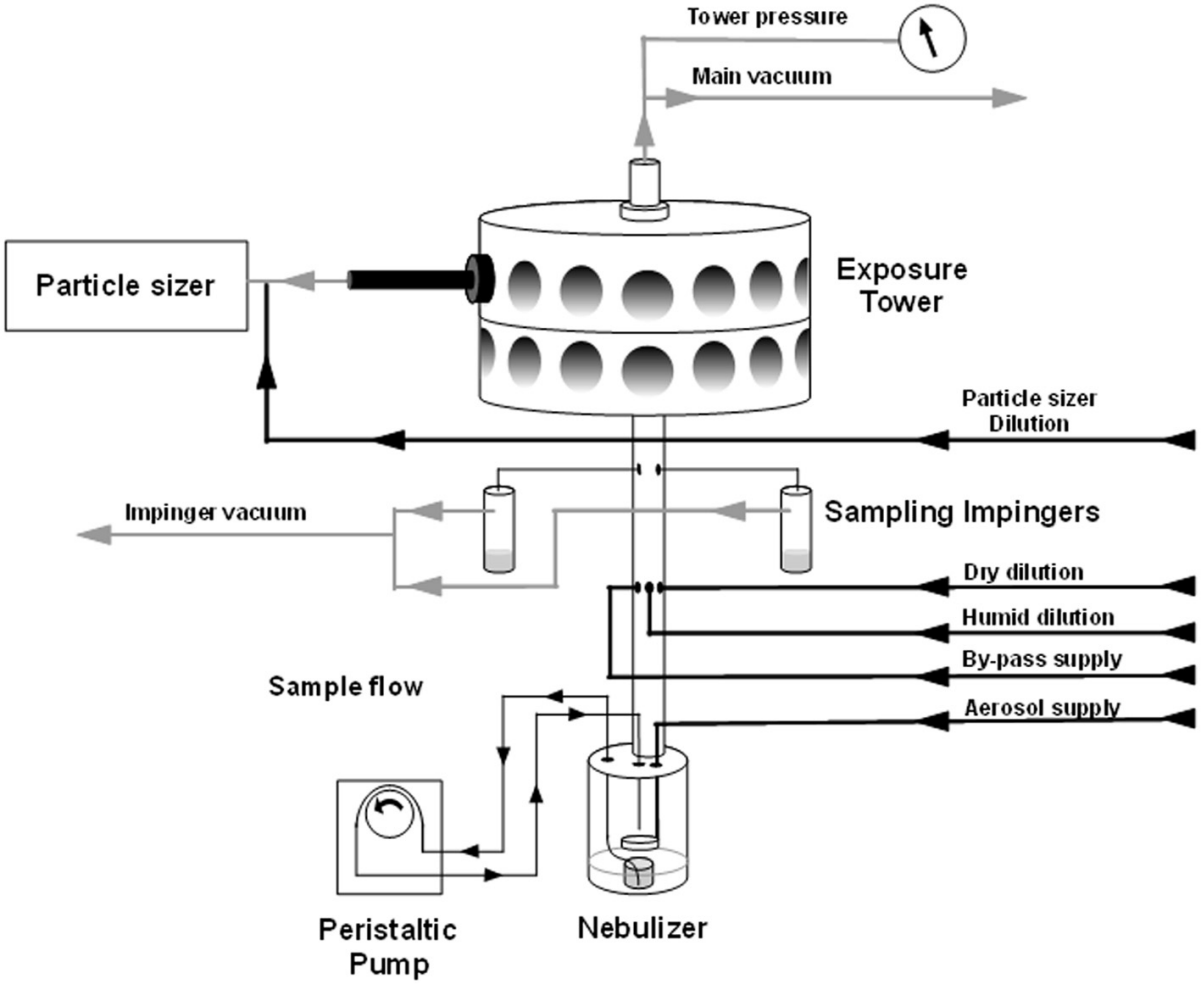
**Nose-only inhalation exposure equipment schematic.** Arrows show direction of supply air and vacuum applied to the exposure tower. Bacterial suspension is drawn from the nebulizer jar via peristaltic pump and allowed to drip on a frit where air is forced through the accumulated film. Excess suspension settles back to the bottom of the jar and is continuously drawn up. Sampling impingers are placed above the point where sample mixes with dilution air, but below the exposure tower.

The nose-only inhalation exposure chamber is diagrammed in Figure 1. Aerosols were created using a SLAG (CH Technologies, Westwood, NJ) fitted with a 1″ diameter, 1 mm thick porous stainless steel disc (frit) with 0.5 μm pore size unless otherwise indicated (Mott Corporation; Farmington, CT). The sample was recirculated throughout the exposure via a peristaltic pump delivering 1 mL/min. Air flow through the SLAG was delivered at 10 standard liters per minute (SLPM) and dilution air was added at the volume required to deliver approximately 10 times the estimated respiratory minute volume (MV) per rat times the total number of nose-only ports on the apparatus (Guyton, 1947). Air flow on all supply, exhaust, and sample lines was controlled using individual mass flow controllers (Alicat Scientific, Inc., Tucson, AZ).

Aerosol particle characterization was performed via a Welas Digital 2000 white light spectrometer particle sizer (Palas; Karlsruhe, Germany). 0.5 SLPM was sampled directly from an animal port. Bacterial viability and concentration in the aerosol were tested by plating serial dilutions of samples collected at 5 SLPM in two all-teflon impingers (SKC, Inc.; Eighty Four, PA), containing 10 mL HIB and 20 μL Emulsion B antifoam (Sigma; St. Louis, MO), via a manifold below the exposure tower.

Aerosol concentration (*C*_Aero_) for a determined impinger sample concentration (*C*_Imp_), impinger sample volume (*V*), sampling flow rate (*Q*_Imp_), and time (*t*) was calculated in CFU/mL as follows: *C*_Aero_ = *C*_Imp_ × *V*/*Q*_Imp_ × *t*. The spray factor is the ratio between the starting concentration of the sample (*C*_Neb_) and the *C*_Aero_ as such: Spray factor = *C*_Aero_/*C*_Neb_. The presented dose is the potential aerosolized dose that might be inhaled by any subject, calculated as *C*_Aero_ × MV × *t*.

### Animals

These studies were carried out in strict accordance with the recommendations in the Guide for the Care and Use of Laboratory Animals of the National Institutes of Health and were approved by the Animal Care and Use Committee of the University of Missouri. All efforts were made to minimize suffering of the animals.

Male and female Brown Norway rats (150–250 g), were purchased from Charles River Laboratories (Wilmington, MA) or were bred and raised in barrier facilities at the University of Missouri. During pneumonic plague challenge, rats were maintained in select agent approved biosafety level 3 containment facilities at the University of Missouri. Animals were challenged using a nose-only exposure system consisting of one or two 12-port layers (CH Technologies; Westwood, NJ) within a Class III biological safety cabinet (Germfree; Ormond Beach, FL). Supply air to the nebulizer and dilution lines was supplied by a Jun Air air compressor while exhaust and sampling lines were attached to a vacuum pump (Gast Mfg.; Benton Harbor, MI) housed within the cabinet. The Class III cabinet was maintained at a negative pressure of approximately −1″ wc relative to the laboratory and the exposure tower was maintained at −0.4 to −0.6″ wc relative to the class III cabinet; all pressures were monitored by magnehelic gauges.

For challenges, rats were restrained in nose-only exposure tubes and placed on the inhalation exposure tower (CH Technologies, Westwood, NJ). Animals were allowed to acclimate to the exposure environment and allowed to breathe clean, dry air for 10 min before being exposed to infectious aerosol for 20 min. Animals were then exposed to clean, dry air for an additional 10 min and returned to housing. All infected rats were monitored regularly by assignment of health scores. Animals were euthanized by CO_2_ asphyxiation followed by bilateral pneumothorax, methods approved by the American Veterinary Medical Association Guidelines on Euthanasia. Following infection, animals were either euthanized at the times indicated or observed for up to 14 days for clinical signs of acute disease.

### Enumeration of bacterial load

Following euthanasia, nasal washes were collected by perfusing sterile PBS into the trachea and extruding through the nasal cavity. Lungs and trachea were aseptically removed, separated, and homogenized in sterile PBS, followed by serial dilution and plating in triplicate on HIA or *Yersinia* selective agar for enumeration (Sarovich et al., 2010). Plates were incubated for 48–72 h at 26°C.

### Serum analysis

Prior to challenge, blood was collected from the saphenous vein; post-challenge blood was collected following euthanasia by cardiac puncture. Samples were centrifuged to remove cells. Serum samples were frozen at –80°C and analyzed after testing for the presence of live bacteria. Samples that contained bacteria were sterilized by passage through a 0.22 μm filter prior to analysis.

For serum analysis of corticosterone levels following handling and exposure to PBS, blood was collected from naïve rats from the saphenous vein a week prior to mock exposure to PBS aerosol. Post-mock exposure to PBS aerosol, the same rats were bled from the saphenous vein and serum was analyzed for corticosterone by Enzyme Immuno Assay (EIA) as described below.

### Quantification of IL-6 by ELISA

Bronchoalveolar lavage fluid was collected from rats by flushing the infected lungs with 5 ml of sterile PBS. Samples were frozen at −80°C and analyzed after testing for the presence of live bacteria. Samples that contained bacteria were sterilized by passage through a 0.22 μm filter prior to analysis. Samples were analyzed for IL-6 by ELISA-Max Deluxe (Biolegend, San Diego, CA) according to the manufacturer's directions.

### Quantification of corticosterone by EIA

Serum samples were analyzed for the presence of corticosterone using the Corticosterone EIA (Immunodiagnostic Systems, Scottsdale, AZ) according to the manufacturer's directions.

### Quantification of blood glucose

Serum samples were analyzed for the presence of glucose using the Glucose Assay Kit (Cayman Chemical Company, Ann Arbor, MI) according to the manufacturer's directions.

### Flow cytometry analyses of inflammation

The inferior lobe of the lungs was removed and injected with 2 mg/ml collagenase-dispase (Roche, Indianapolis, IN), homogenized in 2 mg/ml collagenase-dispase followed by incubation at 37°C, 150 rpm for 45 min. Following incubation, lung samples were filtered through a 40 micron filter and red blood cells were lysed with 1× ACK lysis solution. Samples were stained with mouse anti-rat Ly6G-FITC (Abcam, Cambridge, MA) and washed with FACS buffer (1× PBS with 1% FBS). Samples were run on a MoFlo XDP (Beckman Coulter, Brea, CA) and analyzed using FlowJo software (Tree Star, Inc., Ashland, OR).

### Statistical analysis

Data from all replicates were analyzed for statistical significance by One-Way ANOVA using GraphPad Prism (GraphPad Software, La Jolla, CA).

## Results

### Development of aerosolization protocol for *Y. pestis*

**Figure 2:**
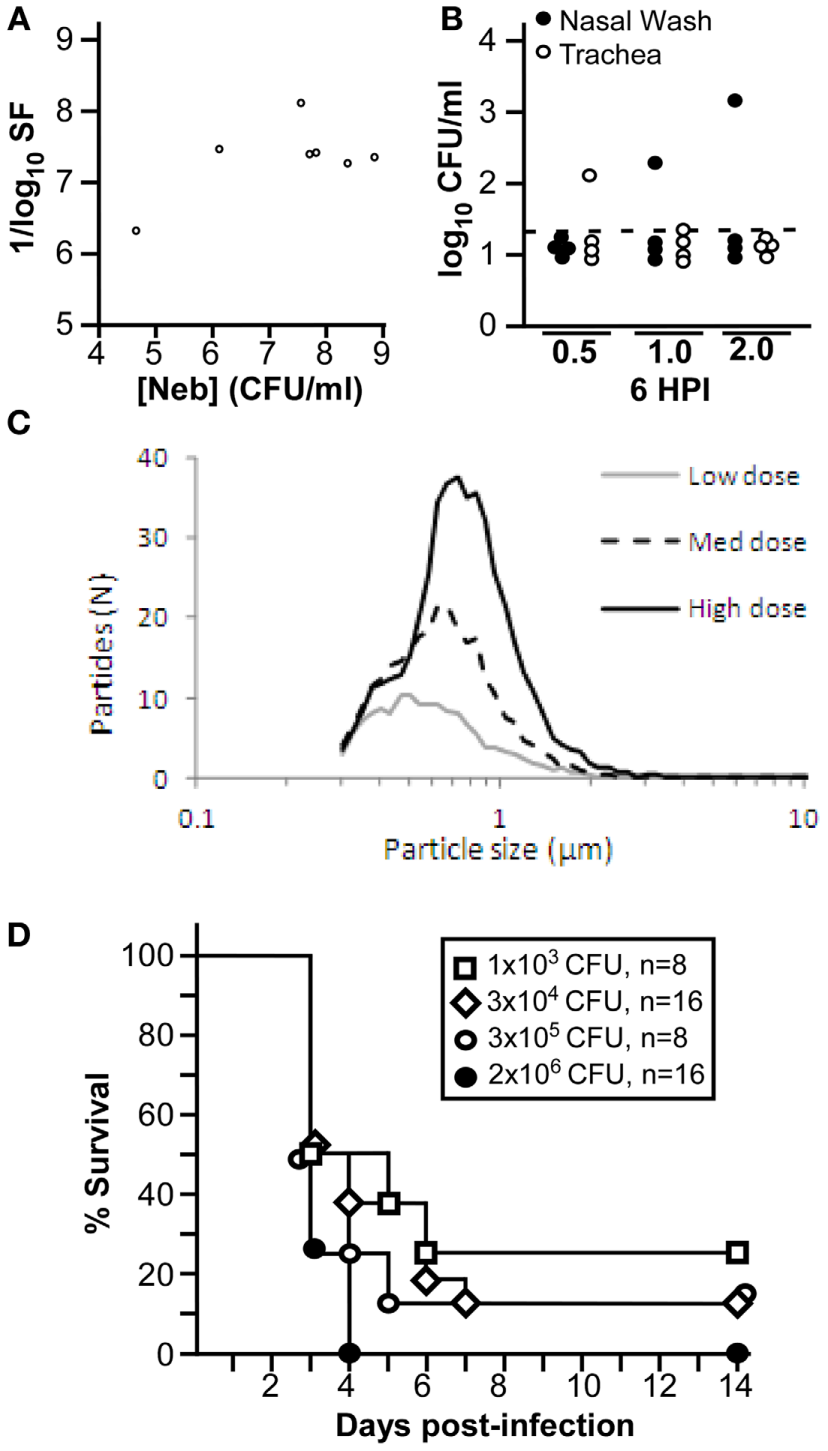
**SLAG nebulizer generates highly infectious *Yersinia pestis* aerosol. (A)** Spray factor is plotted compared to nebulizer concentration for exposure of 8 animals in a single slice chamber. **(B)** Nasal wash and trachea were plated to enumerate bacterial deposition; dotted line indicates limit of detection; *n* = 4 per group, collected in single trial. **(C)** Average particle sizes over the course of three 20 min challenges at low (*C*_neb_ = 1.1 × 10^8^ CFU), medium (*C*_neb_ = 3.9 × 10^8^ CFU) and high (*C*_neb_ = 1.4 × 10^9^ CFU) concentrations were monitored via white light spectroscopy; challenges were performed in a double slice exposure system. All challenge parameters other than nebulizer concentration remained constant during data collection. **(D)** Groups of 8 male or female rats were challenged by inhalation exposure to the indicated doses of *Y. pestis* CO92 and were monitored for 14 days for survival (data shown was combined from 2 independent trials).

*Y. pestis* CO92 was placed in the SLAG nebulizer at different concentrations and aerosolized and collected in impingers containing sterile HIB media. Bacterial concentration in the nebulizer and impingers were determined and the spray factor was calculated. These results showed that spray factor was variable, with higher spray factors typically seen at the lower bacterial concentration and lower spray factors typically observed at the higher bacterial concentrations (Figure 2A). We also measured deposition of bacteria in the upper respiratory tract of the Brown Norway rat of particles generated with different frit sizes (0.5, 1.0, or 2.0 μm). These results demonstrated occasional deposition of bacteria in the trachea but not upper respiratory tract on the 0.5 μm frit while the 1.0 and 2.0 μm frits produced particles that occasionally were found in the nasal passage (Figure 2B). We therefore used the 0.5 μm frit for the remaining experiments.

We challenged male and female Brown Norway rats with increasing doses of *Y. pestis* CO92 delivered by the SLAG nebulizer on a 0.5 μm frit. Aerosol particle size was measured for each dose and found to vary slightly according to the concentration of bacteria in the nebulizer. Overall, the majority of particles found at a low dose (*C*_neb_ = 2.5 × 10^8^ CFU) were 0.5 μm while the majority particle size was 0.7 μm at a 25-fold higher dose (Figure 2C). The range of particles was 0.3–1.1 μm, and overall, these aerosol particles are of the size that would be predicted to be inhaled into the lower respiratory tract (Thomas et al., 2009). The bacteria were highly infectious and rapidly lethal when aerosolized by the SLAG nebulizer, and >50% of animals succumbed to challenge at 72 h post-infection with some animals in each group developing lethal disease in only 60 h (Figure 2D). A presented dose of only 1 × 10^3^ CFU led to 75% lethality while 2 × 10^6^ CFU presented dose caused 100% lethality. These data are comparable to previous reports in the Brown Norway rat using a Collison nebulizer and Madison chamber to model pneumonic plague (Agar et al., 2009).

### Nose-only exposure chamber leads to elevated stress in the brown norway rat

**Figure 3:**
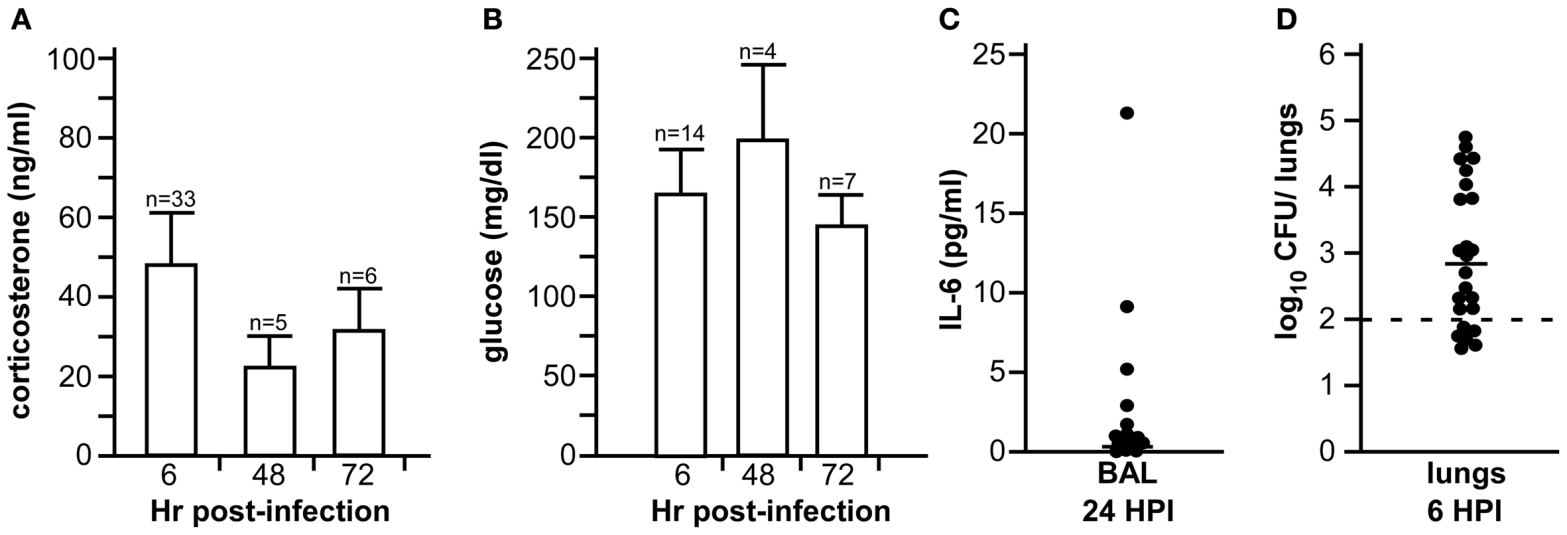
**Bacterial deposition, stress, and pro-inflammatory responses during *Y. pestis* aerosol infection in Brown Norway rats.** Male Brown Norway rats (>225 g) were challenged by inhalation exposure with 5 × 10^5^ CFU *Y. pestis* CO92. Serum corticosterone **(A)** and glucose **(B)** were measured 6, 48, and 72 h post-infection (HPI); **(C)** Bronchoalveolar lavage was collected at 24 HPI and analyzed for IL-6 by ELISA; **(D)** Bacterial titer in lung homogenate was determined at 6 HPI. For **(C)** and **(D)**, bar indicates the median value; in **(D)**, the dotted line indicates the limit of detection. Data shown were collected in one (24 and 72 HPI) or two (6 HPI) independent experiments; *n* = total number of animals studied.

Placement in the nose-only exposure chamber requires significant restraint to optimize inhalation of the bioaerosol through the nares. Since it is widely appreciated that animal restraint causes stress that can be monitored as elevated corticosterone in the serum, we used this measurement to understand how the rats responded to the exposure chamber. We conducted a series of studies to determine deposition of bacteria in the lower respiratory tract while monitoring levels of corticosterone and glucose in the serum. Groups of 8–16 male Brown Norway rats were challenged by inhalation exposure to 5 × 10^5^ CFU *Y. pestis* CO92 (presented dose). Animals were euthanized 6, 24, 48, or 72 h later and blood collected by cardiac puncture. Serum was collected and processed for glucose and corticosterone. Both glucose and corticosterone levels seemed to vary, with a large range of responses (Figures 3A,B). Despite some variations in both corticosterone and glucose levels, differences between all time points were not statistically significant. At the 24 h time point, bronchial lavage fluid was collected and assayed for the presence of the pro-inflammatory cytokine IL-6, which might indicate activation of an NF-κ B inflammatory response. These results showed little to no IL-6 at 24 h post-exposure (Figure 3C). Since *Y. pestis* is known to suppress inflammation in the early stage of infection, this result suggests that the pathogenesis of infection progresses normally in this model. At the 6 h time point, lungs were homogenized in sterile PBS and plated for bacterial enumeration to determine the efficiency of deposition in the lower respiratory tract. Similar to the stress measurements at early time points, inhalation of *Y. pestis* into the lower respiratory tract was variable, ranging from undetectable to 3 × 10^4^ CFU/lung (Figure 3D).

**Figure 4:**
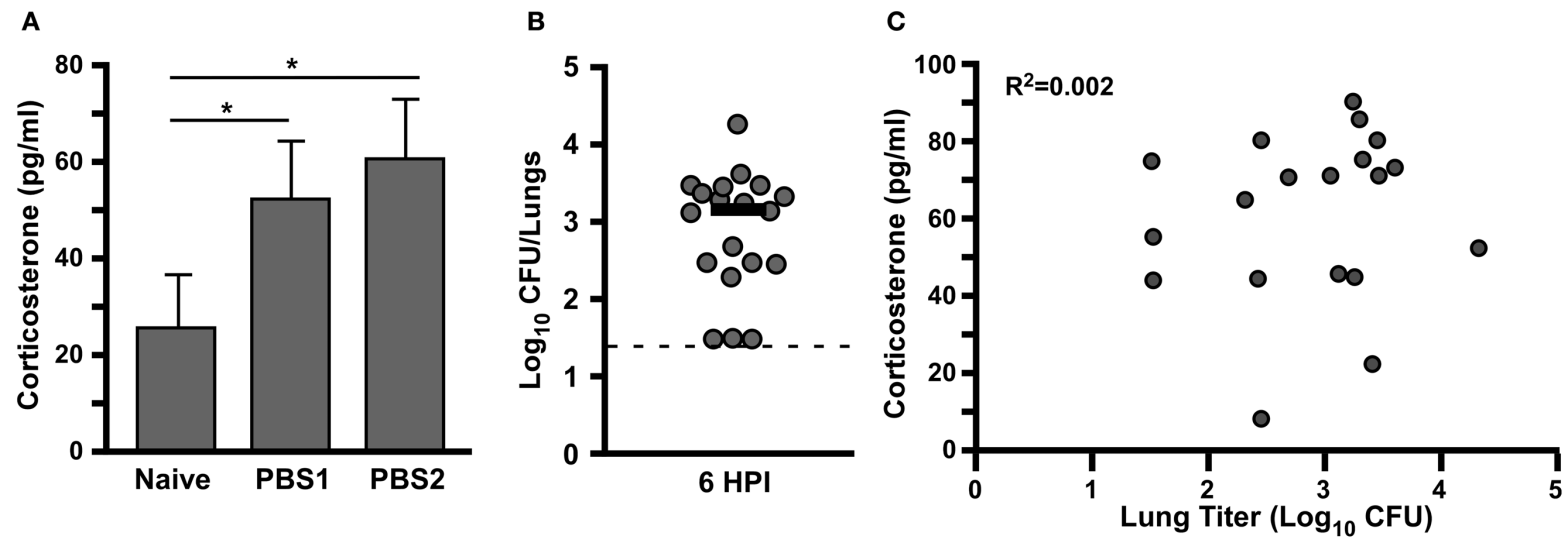
**Elevated serum corticosterone in Brown Norway rats following restraint in nose-only inhalation exposure system does not impact bacterial deposition in the lower respiratory tract. (A)** Male Brown Norway rats were bled from saphenous vein and analyzed for corticosterone on day 0, and following 60 min mock aerosol challenge on day 7 (PBS1) and day 10 (PBS2); mean value is shown, *n* = 18, ^*^p < 0.05. **(B)** On day 13, the same animals were exposed to *Y. pestis* CO92 aerosol (presented dose 5 × 10^5^ CFU) and bacterial titer in the lungs was determined at 6 h post infection (HPI); bar indicates median titer; dotted line indicates limit of detection. **(C)** Correlation analysis for serum corticosterone levels and bacterial titer in the lung at 6 HPI. Data shown was collected in a single trial, *n* = 18.

We monitored the effect of acclimatization on corticosterone in a group of 18 male Brown Norway rats (>225 g). In this experiment, animals were monitored for baseline corticosterone and placed in the exposure chamber for a mock challenge 3 days later. Blood was drawn immediately after removal from the exposure chamber and animals were returned to housing. After another 3 days, animals were once again placed in the chamber and blood drawn immediately after. Animals were then challenged with a presented dose of 5 × 10^5^ CFU and bacterial deposition in the lung was determined at 6 h post-challenge. Elevated corticosterone was observed upon restraint, whether it was the first or second time the animals had been placed in the exposure chamber, and in fact, if anything, the average corticosterone value increased upon the second restraint (Figure 4A). Six hours after exposure, lung deposition was determined and found to range from 5 × 10^1^ to 2 × 10^4^ CFU, a range of deposition from 0.1 to 4% (Figure 4B). No correlation between levels of corticosterone in the serum and deposition of bacteria in the lung was observed (*R*^2^ = 0.002, Figure 4C).

### Rats experience early neutrophil infiltration following aerosol exposure

**Figure 5:**
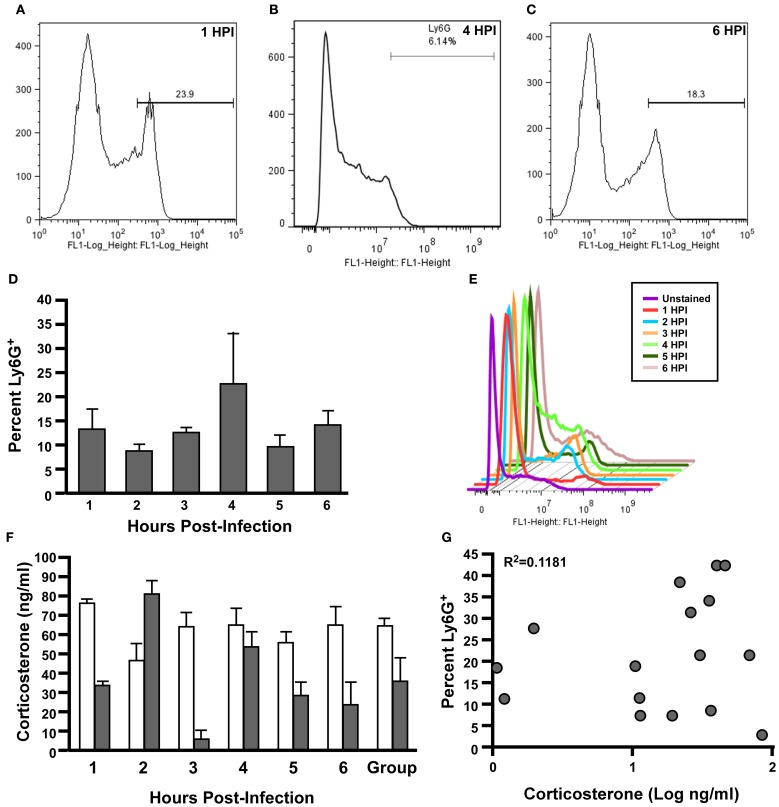
**No correlation between inflammation in the lungs and elevated serum corticosterone following nose-only exposure of Brown Norway rats to *Y. pestis*.** Eighteen Male Brown Norway rats (>225 g) were infected by aerosol challenge with 5 × 10^5^ CFU presented dose of *Y. pestis* CO92. Lungs **(A–E)** and blood **(F)** were harvested at the indicated time points. Lung homogenates were stained for Ly6G^+^-FITC followed by flow cytometric analysis **(A–C)** Representative histograms for 1 HPI, 4 HPI, and 6 HPI; **(D)** Mean percentage Ly6G^+^ staining cells for 1–6 HPI; **(E)** Representative histograms from each time point show the variation of Ly6G^+^ cells in the lung. Data shown are representative of two independent experiments (*n* = 6 total per group). **(F)** Mean serum corticosterone levels from naïve rats (7 days prior to exposure, white bars) and at each time point (gray bars). Data shown is from one replicate, *n* = 3 per group. **(G)** Correlation of percent Ly6G^+^ cells and concentration of corticosterone in the serum at each time point, *R*^2^ = 0.1181. Statistical analysis by One-Way ANOVA with Tukey post-test.

We next tested for recruitment of inflammatory cells to the lung during the first 6 h post-infection to determine whether the restraint-related increase in corticosterone might correlate with a change in inflammation. We monitored baseline corticosterone 3 days prior to nose-only aerosol challenge with 5 × 10^5^ CFU *Y. pestis* CO92. Animals were euthanized every hour for 6 h post-challenge. Serum was collected and lung homogenate was analyzed by flow cytometry for neutrophil recruitment. The inferior lung lobe was homogenized and stained with anti-Ly6G, which is a cell surface marker on neutrophils and eosinophils. We observed Ly6G^+^-staining cell populations in both naïve and challenged animals, ranging from less than 1 to greater than 40% and there were no detectable differences at any time point (Figures 5A–E). We also measured corticosterone in the serum of these animals and observed an increase in the stress hormone at 2 HPI in all animals in the group, consistent with restraint-related stress responses (Figure 5F). We compared neutrophil recruitment and corticosterone levels in each individual animal, combining all time points, and found no correlation but this may be due to the low power of the study (*R*^2^ = 0.1181, Figure 5G). Taken together, the data suggest that restraint-related stress caused by the nose-only exposure chamber does not alter the response to respiratory infection by *Y. pestis*.

## Discussion

New vaccines and therapeutics that target uncommon infectious diseases may be subjected to the US Food and Drug Administration's Animal Rule to obtain licensure. Under this rule, efficacy data in well-characterized animal models may substitute for human efficacy data. In this work, we described an inhalation exposure model of pneumonic plague in the Brown Norway rat and provide additional support that this animal model meets the criteria outlined in the guidance for the Animal Rule (US Department of Health and Human Services Food and Drug Administration, 2009). Aerosolization is the only method that recapitulates natural exposure to respiratory pathogens. Here, we provide a novel method for aerosolizing *Y. pestis* and show the integration of maximum biocontainment with minimal unwanted effects on the experimental model.

Respiratory infections are transmitted by aerosol droplets containing bacterial or viral pathogens. Particles of 1 μm have been reported to be capable of escaping the ciliary action of the trachea and depositing with high efficiency into the lower respiratory tract while particles exceeding 3–5 μm can be trapped in the nasal cavity and/or trachea (Roy et al., 2003; Thomas et al., 2008). In this work, we used the SLAG with frits of increasing diameter pore size to generate aerosols of *Y. pestis* CO92 and deliver through a nose-only exposure system to Brown Norway rats. With this method, we found that pore sizes of 0.5–2.0 μm in most cases generated aerosols with particle sizes of 0.3–2.0 μm, which would likely lead to deposition in the lower respiratory tract.

We observed high virulence of *Y. pestis* CO92 following aerosol infection, with no detectable loss of bacterial viability. A presented dose as low as 1 × 10^3^ CFU caused 75% lethality. Similar nebulizer doses presented to Brown Norway rats through the whole-animal exposure Madison Chamber resulted in similar lethality. Importantly, at all doses, at least 50% of animals succumbed to disease within 60–72 h, some progressing so rapidly that overt signs of disease were not observed. These data indicate that the Brown Norway rat rapidly succumbs to pneumonic plague following aerosol challenge.

Nose-only aerosol exposure is ideal for experimental models of respiratory infections with high morbidity and/or mortality rates. Our exposure chamber takes advantage of maximum containment offered by the negative pressure system and its coordination within a class III biosafety cabinet. This allows the use of animal models with potentially high resemblance to natural exposure, improving the utility of data generated in these comparative models of infectious disease and immunity. Unfortunately, these highly sophisticated and precise instruments involve the introduction of a potential experimental variable: animal restraint. Indeed, our evidence suggests that animals are experiencing elevated stress as a result of the restraint imposed by the exposure chamber. Further, we found that repeated placement of the animals in the exposure chamber did not readily reduce their stress. Though we did not analyze the effect of even more acclimitization to the exposure chamber, we presume that eventually, the animals would acclimate to these conditions. Nevertheless, we found no detectable differences to the progression of the infection imposed by those animals experiencing stress responses indicating extensive acclimatization to the chamber may not be a necessary practice, at least for pneumonic plague models. This may be due to the unique pathogenesis of *Y. pestis*, which establishes an immune-suppressive environment upon pulmonary infection.

SLAG may generally be preferable to the Collison nebulizer for the aerosolization of infectious agents. Previously, the Collison nebulizer has been shown to generate 1–2 μm particles that are effectively inhaled by rodents in a nose-only exposure chamber. The Collison works by capillary action, rapidly accelerating pathogen into a fast moving stream of air with limited ability to adjust air flow rates. Aerosols are created when droplets are formed as the stream impacts the side of the nebulizer jar. In contrast, with the SLAG, different frit pore sizes allow the fine-tuning of particle size. The SLAG allows independent adjustments of air and liquid flow rates. Furthermore, the SLAG can be configured to slowly re-circulate the liquid culture, in contrast to the Collison, allowing a more efficient use of culture such that lower volumes can be used. Finally, the SLAG maintains viability of bacteria whereas, for some bacteria, viability has been shown to decrease by more than 50% during aerosol generation by the Collison nebulizer. Not only is the viability of some infectious agents affected by the forcible treatment in the Collison, but it is likely that surviving cells undergoing this treatment may experience changes in gene expression that could impact virulence. Given the importance that animal models recapitulate the natural infection as much as possible, and the ability to use nose-only exposure chambers with animals that are not anesthetized, these models offer powerful tools for the assessment of efficacy of vaccines and therapeutics against human infection.

### Conflict of interest statement

The authors declare that the research was conducted in the absence of any commercial or financial relationships that could be construed as a potential conflict of interest.
